# Structural and Mechanistic Insights into Dual Cholinesterase Inhibition by Marine Phytohormones

**DOI:** 10.3390/md24010035

**Published:** 2026-01-09

**Authors:** Kumju Youn, Legie Mae Soriano, Mira Jun

**Affiliations:** 1 Department of Food Science and Nutrition, Dong-A University, Busan 49315, Republic of Korea; kjyoun@dau.ac.kr; 2 Center for Food & Bio Innovation, Dong-A University, Busan 49315, Republic of Korea; 3 Department of Health Sciences, The Graduate School of Dong-A University, Busan 49315, Republic of Korea; 2473012@donga.ac.kr

**Keywords:** Alzheimer’s disease, marine phytohormones, cholinesterase inhibitors, enzyme kinetics, molecular docking, molecular dynamic simulation, density functional theory

## Abstract

Cholinergic dysfunction is a hallmark of Alzheimer’s disease (AD), driven by elevated acetylcholinesterase (AChE) and butyrylcholinesterase (BChE) activity that depletes acetylcholine and contributes to amyloid pathology. Current AD treatments face major challenges, including poor brain penetration, short effect duration and safety concerns, highlighting the need for compounds suitable for preventive or earlier-stage intervention. This study investigated marine phytohormones as modulators of cholinergic imbalance, using an integrative strategy encompassing enzymatic assays, QSAR and DFT calculations, molecular docking, molecular dynamics (MD) simulations, and ADMET profiling. Among them, isopentenyl adenine (IPA) and abscisic acid (ABA) showed inhibitory activity against cholinesterases. IPA inhibited both AChE and BChE through distinct mechanisms with noncompetitive inhibition of AChE and competitive inhibition of BChE, while ABA showed selective noncompetitive inhibition of AChE. DFT-based analysis revealed distinct electronic properties supporting differential reactivity. Moreover, IPA interacted with both catalytic and peripheral residues in AChE, and aligned with BChE’s active site, while ABA was bound more peripherally. MD simulations confirmed complex-specific conformational stability based on RMSD, RMSF, Rg, and hydrogen bonding analysis. Both compounds showed low off-target potential against serine proteases and favorable predicted ADMET profiles. These results support the potential of marine phytohormones as preventive modulators of cholinergic dysfunction in AD.

## 1. Introduction

Alzheimer’s disease (AD) is the most prevalent neurodegenerative disorder worldwide, affecting an estimated 57 million people, with numbers projected to exceed 139 million by 2050 primarily driven by global population aging [1,2]. It is characterized by progressive cognitive decline and irreversible neuronal loss, posing a major public health and socioeconomic burden. Despite extensive research, the multifactorial pathogenesis of AD remains incompletely understood [3].

A central pathological feature of AD is the extracellular aggregation of amyloid-beta (Aβ) peptides, which form neurotoxic plaques that impair synaptic signaling, induce oxidative stress, and promote sustained neuroinflammation [4]. In parallel, the intracellular accumulation of hyperphosphorylated tau protein results in the formation of neurofibrillary tangles, disrupting cytoskeletal integrity and axonal transport [5]. These hallmark lesions disrupt synaptic function and contribute to neurotransmitter imbalances, particularly within the cholinergic system [6]. Cholinergic neurotransmission is particularly disrupted in early AD, where overactive AChE and BChE accelerate acetylcholine breakdown, leading to cognitive decline [7].

Beyond serving as downstream targets of AD pathology, AChE and BChE themselves have emerged as active contributors to multiple neurodegenerative processes associated with disease progression. AChE is known to interact with Aβ at its peripheral anionic site (PAS), thereby accelerating Aβ aggregation [8]. In addition, AChE has been reported to directly bind presenilin-1 (PS-1), a catalytic subunit of the γ-secretase complex responsible for Aβ generation, further amplifying plaque formation [6,9]. Disruption of central cholinergic signaling has also been implicated in tau hyperphosphorylation, neuroinflammation, apoptosis, and neurotransmitter imbalances, reinforcing the multifaceted pathological roles of cholinesterases [10]. This multiple involvement in both neurotransmitter degradation and Aβ fibrillogenesis makes cholinesterases compelling targets for therapeutic intervention. Although cholinergic dysfunction is not considered the earliest pathological event in AD, it plays a critical role in mediating the clinical manifestations of memory decline and synaptic failure, making it a central therapeutic target [11].

Cholinesterase inhibitors (ChEIs) exert their therapeutic effects by increasing ACh levels at synaptic junctions, thereby temporarily enhancing cholinergic transmission. Current inhibitors such as donepezil, rivastigmine, and galantamine exhibit limitations including dose-dependent side effects, low target selectivity, restricted blood–brain barrier (BBB) permeability, and eventual loss of efficacy with prolonged administration [12,13]. These limitations highlight the urgent need to identify novel ChEIs with improved safety profiles and broader therapeutic mechanisms.

In the search for alternative molecular scaffolds, marine algae are increasingly recognized as reservoirs of pharmacologically active small molecules, including phytohormones with diverse structural and functional attributes. These endogenous signaling compounds, traditionally studied in terrestrial plants, have also been identified across multiple algal lineages, where they modulate physiological responses such as growth, reproduction, and stress tolerance. Phytohormones derived from marine algae including cytokinins and sesquiterpenoid acids represent a class of structurally diverse metabolites that remain largely unexplored for neurological applications [14]. These molecules, while primarily known for their roles in plant physiology and stress adaptation, possess physicochemical properties compatible with central nervous system (CNS) drug development. While phytohormones are not conventionally regarded as drug candidates, their low molecular weight, structural diversity, and functional reactivity render them suitable scaffolds for bioactivity screening [15,16]. Given the pressing need for novel ChEIs to combat neurodegenerative disorders such as AD, these algal metabolites represent an underexplored chemical space for CNS-targeted drug discovery.

To explore the therapeutic relevance of marine algal phytohormones as novel ChEIs, a mechanism-driven, multi-step investigation was undertaken. Computational profiling and drug-likeness filtering were applied to identify candidates with physicochemical properties compatible with CNS targeting. Electronic structure analyses provided insight into molecular reactivity and interaction potential. Selected compounds were then subjected to in vitro enzymatic evaluation to assess their inhibitory activity and underlying mechanisms against AChE and BChE. To further elucidate binding specificity and mode of action, molecular docking was conducted at both catalytic and peripheral enzyme sites, followed by molecular dynamics (MD) simulations to examine the structural stability and dynamic behavior of the ligand–enzyme complexes. This integrative approach was designed to uncover not only functional inhibition but also mechanistic and structural insights, supporting the potential of marine phytohormones as scaffolds for therapeutic development in neurodegenerative diseases.

## 2. Results

### 2.1. Chemical Structures and QSAR Descriptor Analysis of Phytohormones from Marine Algae

**Figure 1:**
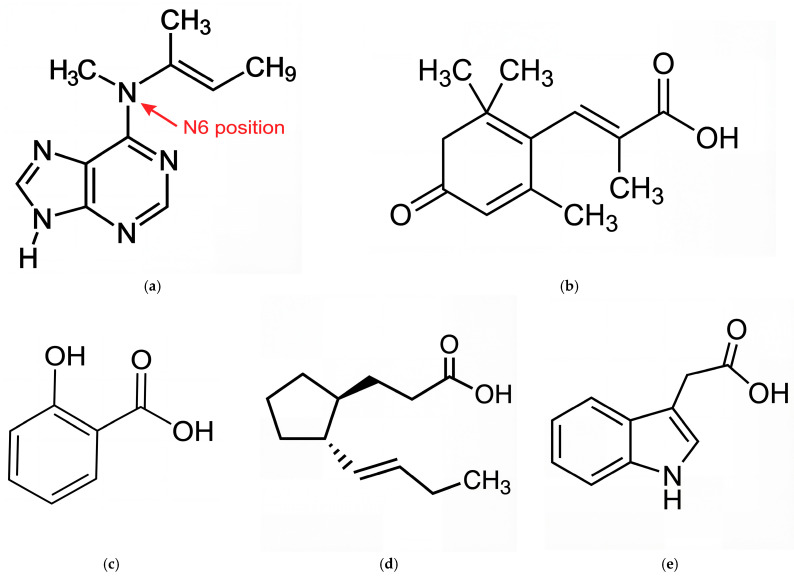
Chemical structures of selected phytohormones: (**a**) isopentenyl adenine (IPA) with the N^6^ position on the adenine moiety indicated by an arrow, (**b**) abscisic acid (ABA), (**c**) salicylic acid (SA), (**d**) jasmonic acid (JA), and (**e**) indole-3-acetic acid (IAA).

Figure 1 presents the structures of isopentenyl adenine (IPA), abscisic acid (ABA), salicylic acid (SA), jasmonic acid (JA), and indole-3-acetic acid (IAA), which have been reported in diverse marine algal species, where they regulate growth and mediate responses to environmental stimuli.

**Figure 2:**
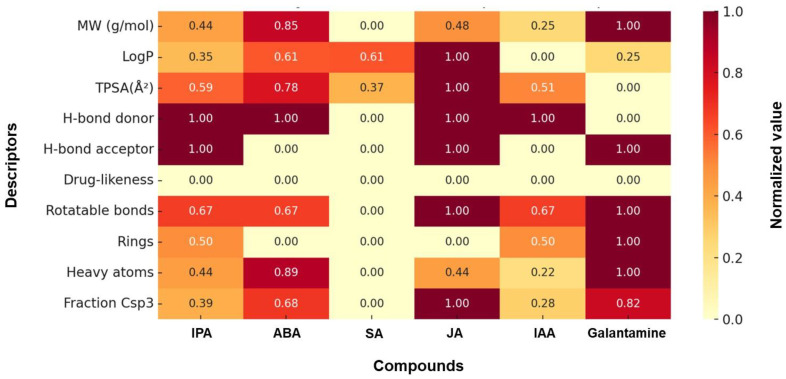
Heatmap visualization of normalized physicochemical descriptors for phytohormones and galantamine.

To evaluate their drug-like potential, a set of widely recognized physicochemical descriptors was calculated (Table 1 and Figure 2). These descriptors, including molecular weight (MW), lipophilicity (LogP), topological polar surface area (TPSA), hydrogen bond donors/acceptors, and the fraction of sp^3^-hybridized carbons (Csp^3^), are widely applied in QSAR modeling and ADMET prediction. In the context of CNS drug development, they serve as indicators of membrane permeability, metabolic stability, and BBB penetration potential. Galantamine was used as the reference compound because its physicochemical profile represents a combination of molecular size, polarity, and hydrogen-bonding capacity that has been demonstrated to support sufficient oral bioavailability and CNS exposure in clinical use [17]. Therefore, comparison against galantamine allows assessment of whether marine phytohormones fall within a physicochemical space compatible with effective cholinesterase targeting in the CNS, rather than relying solely on theoretical descriptor thresholds.

All five phytohormones met basic drug-likeness criteria including MW < 500, LogP < 5, TPSA ≤ 140 Å^2^, and H-bond donor/acceptor rules. Among them, IPA and ABA showed the closest overall descriptor profiles to galantamine, particularly in LogP, TPSA, and H-bonding capacity. Figure 2 illustrates a heatmap of normalized descriptor values across all six compounds, providing a visual summary of their multidimensional physicochemical profiles. Compounds with similar color intensities across key descriptors were interpreted as exhibiting higher overall similarity. Based on this clustering pattern, IPA and ABA showed the closest alignment to galantamine, particularly in parameters related to polarity, size, and hydrogen-bonding capacity. This heatmap-based comparison supports their selection for further analysis, consistent with prioritization strategies commonly employed in drug discovery [18,19]. In this analysis, IPA and ABA clustered closest to galantamine, especially in polarity-related and molecular size parameters, supporting their selection for further evaluation. Conversely, SA and IAA showed lower overall similarity to galantamine, particularly in terms of drug-likeness (Csp^3^ fraction and rotatable bonds), which may limit their bioavailability or target affinity. JA shared partial similarities in specific features (e.g., LogP, Csp^3^), but did not align comprehensively across the descriptor set. Overall, based on the descriptor-based comparison, IPA and ABA are the most promising phytohormones for further in silico and in vitro evaluation as ChEIs.

### 2.2. DFT-Based Electronic Property Analysis of Isopentenyl Adenine (IPA) and Abscisic Acid (ABA)

To elucidate whether intrinsic quantum chemical properties can effectively predict biological binding behavior of selected ligands with cholinesterase enzymes, a comparative analysis between density functional theory (DFT)-derived electronic descriptors was performed. Key descriptors, including highest occupied molecular orbital (HOMO) and lowest unoccupied molecular orbital (LUMO) energies, energy gaps (ΔE), dipole moments, and reactivity indices, were evaluated to assess molecular stability and potential reactivity (Table S1 and Figure S1).

IPA presented a notably balanced electronic profile, with a HOMO energy of −6.09 eV and a LUMO energy of −0.90 eV, resulting in a HOMO-LUMO energy gap (ΔE) of 5.19 eV. This gap suggests a molecule that is electronically stable, yet still flexible enough to engage in productive interactions. Most strikingly, it exhibited a dipole moment of 7.53 Debye, significantly surpassing those of the other ligands, indicating pronounced molecular polarity and a strong capacity for electrostatic interactions in a polar biological environment.

In comparison, ABA presented the narrowest energy gap of 4.51 eV (HOMO = −6.74 eV, LUMO = −2.22 eV), reflecting enhanced chemical softness and increased electron delocalization. However, its dipole moment (2.25 Debye) was markedly lower, implying reduced capacity for directional interactions. Interestingly, despite its high electrophilicity index (ω = 4.45 eV), its lower polarity may limit its interaction versatility. Galantamine, a well-known cholinesterase inhibitor, displayed a balanced electronic profile, with a HOMO-LUMO gap of 5.09 eV and the lowest dipole moment (2.04 Debye) among the tested compounds.

Collectively, these observations underscore how DFT-calculated dipole moments, in particular, serve as a reliable predictor of molecular interaction diversity in enzyme-ligand systems. While HOMO-LUMO energy gaps and electrophilicity indices provide valuable insights into chemical reactivity, dipole moments emerged as a more reliable indicator of binding performance in enzyme-ligand complexes. These findings highlight dipole moment as a key electronic predictor in cholinesterase-ligand interactions, reinforcing the utility of DFT-based pre-screening in rational inhibitor design.

### 2.3. In Vitro Cholinesterase Inhibition, Mechanism and Specificity of IPA and ABA

The inhibitory potential of five marine phytohormones as cholinesterase modulators was evaluated through enzyme-based screening, followed by kinetic analysis to determine inhibition constants (Ki) and elucidate the underlying mechanisms. The kinetic models employed included Dixon and Lineweaver–Burk plots, along with secondary replots of Km and Vmax, which allowed classification of competitive and non-competitive inhibition.

Among the tested compounds, only IPA and ABA showed potent cholinesterase inhibition, whereas SA, JA, and IAA exhibited no significant activity at concentrations up to 100 μM and were thus excluded from further kinetic analysis. IPA inhibited both AChE and BChE, with IC_50_ values of 40.52 ± 0.71 μM and 25.81 ± 0.05 μM, respectively, indicating dual activity across isoforms. In contrast, ABA demonstrated moderate inhibition restricted to AChE (IC_50_ = 76.29 ± 2.94 μM), suggesting enzyme selectivity (Table 2).

**Figure 3:**
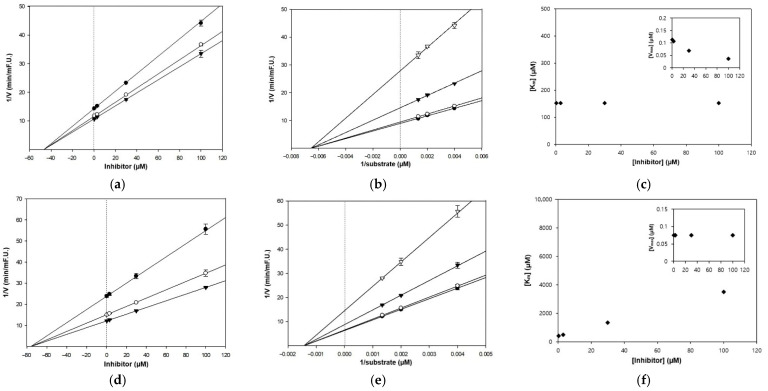
Kinetic analysis of AChE inhibition by IPA and ABA. Panels (**a**–**c**) represent the inhibition of AChE by IPA, as analyzed by (**a**) Dixon plot, (**b**) Lineweaver–Burk plot, and (**c**) secondary replots of Km and Vmax. Panels (**d**–**f**) correspond to AChE inhibition by ABA, showing (**d**) Dixon plot, (**e**) Lineweaver–Burk plot, and (**f**) secondary replots of kinetic parameters. In the Dixon plot (**a**,**d**), symbols represent different substrate concentrations (S = 250, 500, and 750 µM). In the Lineweaver–Burk plot (**b**,**e**), symbols represent different concentrations of inhibitor (I = 0.3, 3, 30, and 100 µM).

**Figure 4:**
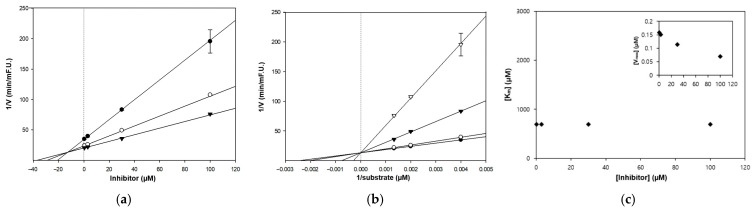
Kinetic analysis of BChE inhibition by IPA using (**a**) Dixon plot, (**b**) Lineweaver–Burk plot, and (**c**) secondary replots of kinetic parameters (Km and Vmax). In the Dixon plot (**a**), symbols represent different substrate concentrations (S = 250, 500, and 750 µM). In the Lineweaver–Burk plot (**b**), symbols represent different concentrations of inhibitor (I = 0.3, 3, 30, and 100 µM).

Subsequent kinetic analyses revealed isoform-specific inhibitory mechanisms for IPA. With AChE, a non-competitive mode was observed, characterized by a reduction in Vmax without changes in Km, suggesting binding at an allosteric site rather than the catalytic center. The corresponding Ki was determined to be 46.8 μM, indicating a moderate affinity toward the non-catalytic pocket. In contrast, the inhibition of BChE followed a typical competitive model. The Lineweaver–Burk plots showed increasing Km values while Vmax remained unaffected, indicating that IPA competes directly with the substrate at the active site. These kinetic models and isoform-specific interactions are illustrated in Figure 3 and Figure 4.

Although less potent than IPA, ABA followed a similar allosteric inhibition pattern toward AChE. A progressive reduction in Vmax was observed while Km remained constant, consistent with an allosteric binding mechanism (Table 2 and Figure 3). The Ki was calculated at 76.3 μM, suggesting relatively weaker interaction compared to IPA. No measurable inhibition against BChE was detected, and further kinetic modeling was not performed.

Neither IPA nor ABA exhibited appreciable inhibition of unrelated serine proteases, with all off-target effects remaining below 18% even at high concentrations (100 μM), supporting their selectivity for cholinesterases (Table 3).

### 2.4. Molecular Docking and Pharmacokinetic Profiling of IPA and ABA

**Figure 5:**
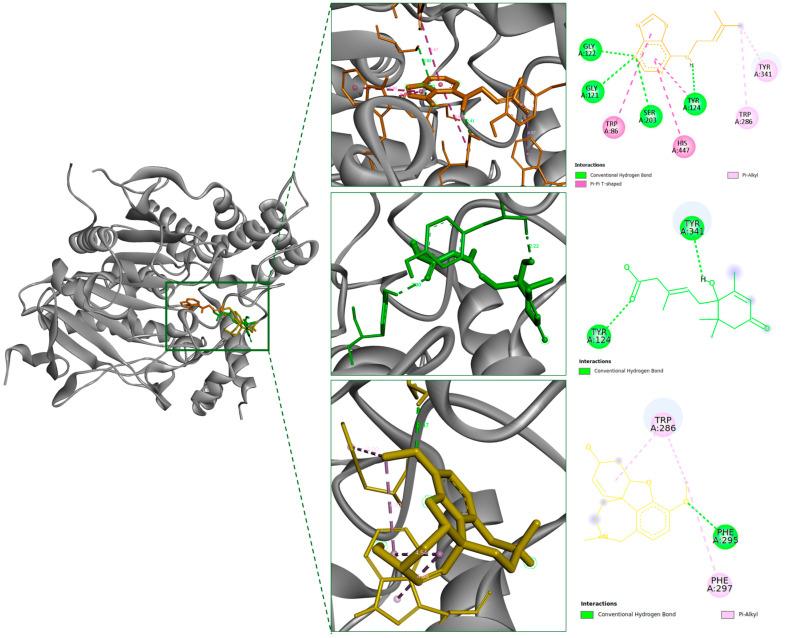
Three-dimensional and two-dimensional interaction diagrams of IPA, ABA, and the reference inhibitor *galantamine* docked with AChE. The ligands are shown in orange (IPA), green (ABA), and yellow (galantamine). The 3D binding pocket views depict the spatial orientations of the ligands primarily within the PAS of AChE, while the 2D interaction maps highlight key residue interactions including hydrogen bonds and π–alkyl or π–π stacking contacts.

Based on their confirmed in vitro activity, IPA and ABA underwent molecular docking analysis to investigate their interaction modes with AChE and BChE at the residue level. As shown in Figure 5 and Table 4, IPA exhibited the most favorable binding energy toward AChE, mediated through multiple hydrogen bonds and π–π/hydrophobic interactions spanning both the catalytic site and PAS. This multi-domain engagement supports its experimentally observed non-competitive inhibition and highlights a broad interaction surface across the enzyme gorge. Its docking energy was more favorable than galantamine (−7.39 kcal/mol), suggesting favorable interaction strength under comparable conditions.

In contrast, ABA displayed a moderate binding affinity toward AChE, with a docking score of −7.04 kcal/mol. Its binding mode revealed two hydrogen bonds formed with Tyr124 and Tyr341, both situated near the PAS. Hydrophobic contacts were also observed at the enzyme surface; no direct interactions were identified with residues belonging to the catalytic triad. Compared to IPA, which engaged both peripheral and catalytic residues, ABA exhibited a more spatially restricted binding pattern within AChE, consistent with its moderate non-competitive inhibition.

**Figure 6:**
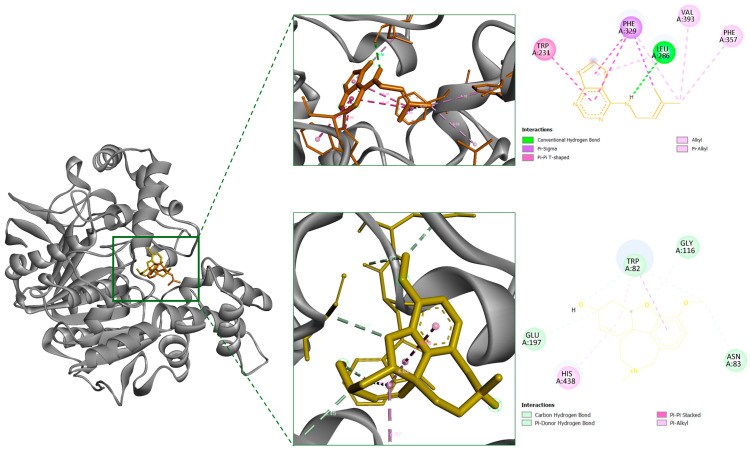
Three-dimensional and two-dimensional interaction diagrams of IPA and the reference inhibitor galantamine docked with BChE. The ligands are shown in orange (IPA) and yellow (galantamine). The 3D binding pocket views depict their conformations and interactions primarily within the catalytic active site (CAS) of BChE, consistent with competitive inhibition. The accompanying 2D interaction maps illustrate key residue contacts, including hydrogen bonding, π–π stacking, and hydrophobic interactions.

For BChE, the docking energy of IPA was found to be −7.26 kcal/mol. It formed a hydrogen bond with Leu286 and established additional π- and hydrophobic interactions with Trp231, Leu286, Phe329, Phe357 and Val393 (Figure 6 and Table 4). These residues are positioned within or adjacent to the enzyme’s catalytic site, which is consistent with the experimentally determined competitive inhibition profile.

The pharmacokinetic and toxicity profiles of IPA and ABA were evaluated using in silico ADMET predictions (Table 5). Both compounds exhibited excellent human intestinal absorption and favorable BBB permeability, indicating their potential to reach central nervous system targets. Neither compound was identified as a substrate or inhibitor of P-glycoprotein (P-gp), suggesting a low risk of efflux-mediated bioavailability loss. In addition, no hERG channel inhibition was predicted, which supports their potential safety as CNS-active ChEIs.

### 2.5. Molecular Dynamic Simulation and Binding Free Energy Analysis of IPA–AChE, ABA–AChE, and IPA–BChE Complexes

To investigate the structural impact of ligand binding at the atomic level, MD simulations were conducted over a 100 ns trajectory. This analysis expanded upon docking-based predictions by capturing time-dependent conformational behaviors of each complex. Backbone root mean square deviation (RMSD), residue-wise root mean square fluctuation (RMSF), hydrogen bond occupancy, and radius of gyration (Rg) were monitored to assess global stability, local flexibility, interaction persistence, and structural compactness, respectively. To further quantify binding strength, MM/PBSA calculations were performed on the equilibrated MD trajectories to estimate the binding free energies and their energetic components.

**Figure 7:**
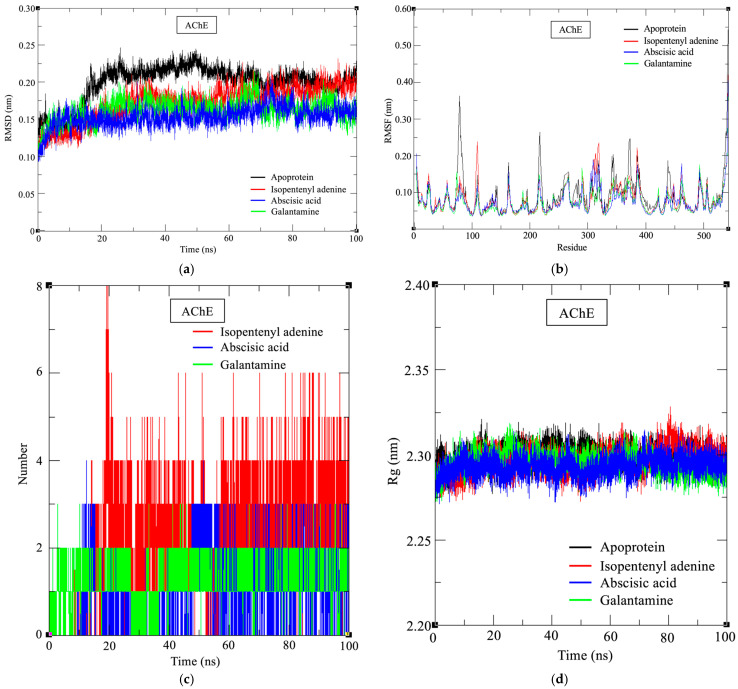
Molecular dynamics trajectories of the IPA–AChE and ABA–AChE complexes over a 100 ns simulation period. (**a**) Time evolution of backbone root mean square deviation (RMSD) of Cα atoms over a 100 ns simulation. (**b**) Root mean square fluctuation (RMSF) per residue of backbone atoms. (**c**) Time-dependent profile of hydrogen bonds formed between ligand and AChE. (**d**) Radius of gyration (Rg) calculated over the simulation period.

All AChE-bound complexes remained structurally stable throughout the simulation. IPA–AChE showed an average RMSD of 0.172 ± 0.024 nm, slightly higher than ABA–AChE (0.162 ± 0.015 nm), yet lower than the apo enzyme (0.198 ± 0.026 Å), indicating stabilization with retained flexibility (Figure 7a). Interestingly, these values were similar to those of the galantamine–AChE complex (0.153 ± 0.013 nm), which was included as a positive control. This suggests that IPA and ABA provide a comparable level of structural stabilization. RMSF analysis confirmed this trend, with IPA and ABA reducing residue-level fluctuations relative to apo-AChE (IPA: 0.081 nm, ABA: 0.073 nm, apo: 0.089 nm, Figure 7b). Notably, peak fluctuations were observed at residue 542 (IPA: 0.420 nm, ABA: 0.393 nm, apo: 0.543 nm), suggesting that ligand binding dampens local motion, particularly in flexible loop regions. These results were comparable to the galantamine–AChE complex (RMSF: 0.074 nm; residue 542: 0.404 nm), supporting the potential of IPA and ABA to confer similar dynamic stabilization.

As illustrated in Figure 7c, hydrogen bonding patterns further differentiated the two complexes. IPA–AChE maintained an average of 2.11 hydrogen bonds, with high temporal persistence. Galantamine–AChE formed ~0.97 hydrogen bonds on average, showing moderate polar interaction compared to IPA. In contrast, ABA–AChE averaged only 0.84, indicating weaker and less frequent polar contacts. Rg remained stable across all AChE systems (IPA: 2.297 ± 0.007 nm, ABA: 2.295 ± 0.006 nm, apo: 2.299 ± 0.006 nm), indicating that both IPA and ABA maintained global compactness comparable to the positive control (1.870 ± 0.089 nm), reinforcing their dynamic stability (Figure 7d).

**Figure 8:**
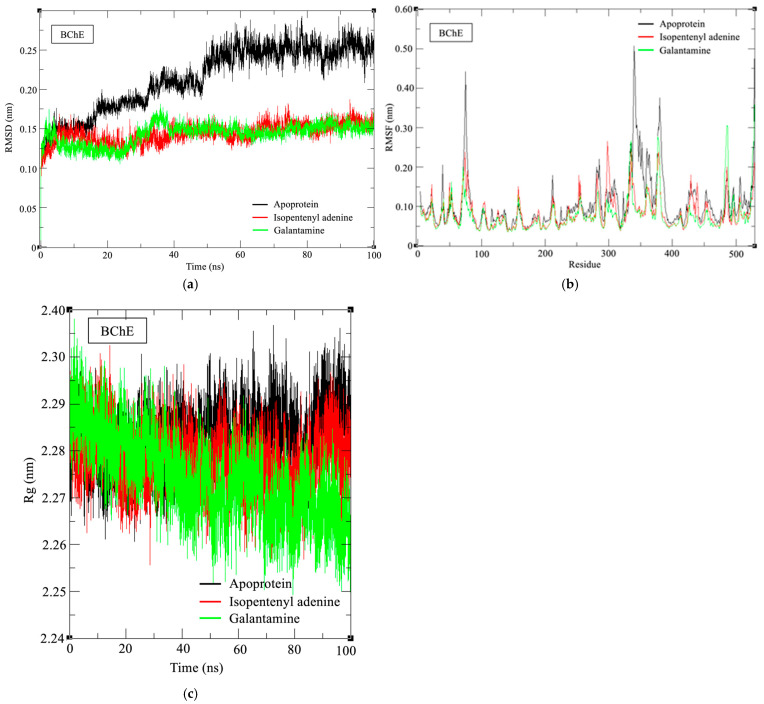
Molecular dynamics trajectories of the IPA–BChE complex and apo-BChE. (**a**) Backbone RMSD of Cα atoms over 100 ns for ligand-bound and apo forms. (**b**) Residue-wise RMSF of backbone atoms. (**c**) Radius of gyration (Rg) plotted over simulation time.

For BChE, the RMSD dropped to 0.143 ± 0.012 nm, a marked decrease compared to apo-BChE (0.214 ± 0.041 nm, Figure 8). RMSF also declined to 0.078 nm, with maximum fluctuation observed at residue 75 (0.298 nm), significantly lower than the apo peak at residue 340 (0.507 Å). A similar stabilizing trend was observed for galantamine–BChE (RMSD: 0.143 ± 0.013 nm; RMSF: 0.077 nm). Rg values remained virtually unchanged (IPA: 1.801 ± 0.083 Å; apo: 2.283 ± 0.006 Å), indicating maintenance of global compactness. Notably, no persistent hydrogen bonds were observed, which is consistent with the idea that hydrophobic and π–interactions, rather than polar contacts, may dominate the competitive binding mode in BChE.

Consistent with the dynamic stability observed in the MD simulations, MM/PBSA analysis revealed that IPA exhibited a more favorable total binding free energy (ΔG_TOTAL) than ABA in AChE complexes (Table 6). This difference was primarily driven by stronger van der Waals and electrostatic contributions. Galantamine showed the most favorable binding energy overall, consistent with its role as a reference inhibitor. In BChE, IPA binding was likewise dominated by van der Waals interactions with limited electrostatic contribution, reflecting the hydrophobic nature of the BChE binding gorge. The resulting binding free energy of the IPA–BChE complex was comparable to that of galantamine–BChE, supporting a competitive binding mode mediated predominantly by nonpolar interactions rather than persistent hydrogen bonding.

## 3. Discussion

Advances in blue biotechnology have enabled the discovery of diverse bioactive compounds from seaweeds and microalgae, including phytohormones with potential pharmacological relevance [14]. IPA is a bioactive derivative of the cytokinin class that promotes cellular growth and differentiation under favorable conditions [15]. In contrast, ABA is a key stress-related hormone that accumulates under abiotic stresses such as salinity, oxidative damage, and dehydration. It is also among the most abundant stress hormones in marine organisms [16]. However, their potential interactions with mammalian cholinesterases, particularly in the context of neurodegenerative disease, remain insufficiently characterized. In this study, IPA and ABA were identified as cholinesterase-inhibiting agents, pointing to an expanded biological role beyond their established functions in plant and algal systems. This dual experimental–computational approach revealed their latent cholinergic activity, indicating an underexplored pharmacological potential for these marine phytohormones.

AChE and BChE exhibit electrostatically enriched binding environments, composed of charged and polar residues distributed across both the active site and peripheral domains, which influence ligand recognition and inhibition profiles. In this study, quantum chemical descriptors derived from DFT calculations were employed to interpret ligand behavior in conjunction with docking and kinetic data. IPA exhibited a high dipole moment (7.53 Debye) and a moderate HOMO–LUMO gap (5.19 eV), electronic features that align with its experimentally confirmed dual engagement of the catalytic triad and peripheral anionic site, consistent with its non-competitive inhibition profile. ABA, in contrast, showed selective AChE inhibition with a lower dipole moment and narrower energy gap, corresponding to binding confined primarily to the PAS. These electronic distinctions suggest that molecular polarity and orbital distribution influence binding site preference, with high-polarity ligands like IPA displaying broader spatial compatibility compared to the more restricted behavior of ABA. This relationship is further reflected in the findings of Hasan et al. (2023) reported that coumarin–Schiff base inhibitors with elevated dipole moments preferentially targeted PAS residues such as Trp86 and Tyr337, exhibiting non-competitive inhibition mediated by π–π stacking and hydrogen bonding [20]. Their results reinforce our observation that molecular polarity may promote peripheral binding and allosteric modulation in cholinesterase inhibition. Collectively, these findings highlight the utility of electronic descriptors, especially dipole moment and HOMO–LUMO gap, as predictive indicators of binding orientation and inhibitory mode, particularly when evaluated in conjunction with structural and kinetic analyses. Specifically, the high dipole moment of IPA may contribute to its competitive inhibition toward BChE by facilitating favorable ligand orientation during entry into the catalytic gorge, rather than by inducing persistent polar interactions within the binding pocket. Unlike its peripheral binding to AChE, this electronic profile enables IPA to adopt a substrate-like configuration that allows effective access to and occupancy of the BChE catalytic site, despite the predominantly hydrophobic nature of the cavity.

Beyond enzymatic inhibition, ligand binding to PAS of AChE may have functional implications for amyloid-related pathology. In the present study, IPA was found to bind both the PAS of AChE and the catalytic site of BChE, suggesting a dual mechanism that may simultaneously influence cholinergic tone and interfere with Aβ aggregation. This is particularly relevant given that PAS residues such as Trp286 and Tyr341 are known to promote Aβ fibrillogenesis. Our results showed that IPA interacts with these residues, supporting its potential to interfere with aggregation-prone conformations. Natural compounds including berberine derivatives, piceatannol, scopoletin, and ceanothane derivatives have been reported to exert similar effects via PAS-targeted AChE inhibition [21,22,23,24]. In contrast, ABA exhibited non-competitive inhibition with binding primarily localized to the PAS, a finding consistent with its low dipole moment and spatial restriction. While in vivo data on IPA remain limited, structurally related analogs such as N6-isopentenyladenosine have demonstrated enhanced neuronal viability and antioxidant effects in hippocampal models [25], suggesting potential CNS relevance. Similarly, ABA has shown neuroprotective and anti-inflammatory actions in Parkinson’s disease models through microglial suppression and dopaminergic neuron preservation [26], indicating possible therapeutic overlap with AD pathology.

Although most existing studies have centered on AChE inhibition, recent evidence suggests that BuChE becomes increasingly relevant during AD progression. BuChE activity has been reported to increase by up to 90% in certain brain regions, eventually surpassing AChE as the dominant enzyme for acetylcholine hydrolysis in affected cortical areas [27,28]. This enzymatic shift highlights a pathological transition of BChE from a minor to a compensatory cholinesterase, warranting further attention in AD-targeted strategies. In this context, our findings that IPA acts as a BChE inhibitor with competitive binding at the catalytic site provide new mechanistic insight into early intervention potential. Notably, IPA formed hydrophobic and π–π stacking interactions with the residues Trp231 and Phe329, which have been reported as critical for stabilizing the ligand in potent BChE inhibitors [29]. These interactions were consistent with its binding persistence and spatial compatibility within the catalytic gorge. The purine-based scaffold of IPA aligned favorably with the hydrophobic environment of BChE’s active site, underscoring its potential as a lead structure for the development of selective modulators.

Consistent with the distinct physicochemical characteristics of the BChE binding pocket, although IPA possesses a high dipole moment (7.53 Debye), its interaction profile within BChE was dominated by nonpolar contacts. This behavior highlights that the contribution of electronic polarity depends on the physicochemical characteristics of the binding pocket rather than on ligand polarity alone. AChE features a more polar and charged active site that favors hydrogen bonding and electrostatic interactions, whereas BChE presents a broader, more hydrophobic binding gorge, reducing the prevalence of polar contacts. Accordingly, IPA adapted its binding mode to engage in π–stacking and van der Waals interactions in BChE, consistent with the absence of persistent hydrogen bonds observed in the MD simulations. This flexibility underscores how dipole moment reflects a compound’s overall electrostatic potential, which may promote interaction diversity even in hydrophobic environments by supporting adaptable π-mediated interactions. These findings suggest that high dipole moment does not solely predict polar interactions but may also support spatial alignment within hydrophobic cavities through complementary electronic and steric properties. This aligns with previous reports that polar ligands can achieve stable binding in apolar pockets via π-mediated interactions when steric complementarity is satisfied [30].

The differential inhibition profiles of IPA and ABA can be rationalized by integrating their structural characteristics with the stability and binding dynamics of their respective enzyme complexes. As a purine-based cytokinin, IPA consists of an adenine core substituted with an isopentenyl side chain at the N^6^ position. IPA’s molecular architecture contributes not only to spatial complementarity within the cholinesterase active site but also to conformational stability during binding, as demonstrated by MD simulations. The adenine moiety formed persistent π–π stacking interactions with catalytic residues such as Trp86 and His447, which remained stable throughout the 100 ns trajectory, while the isopentenyl side chain maintained π–alkyl contacts with peripheral residues including Tyr341 and Trp286. This extended binding network was reflected in consistently low RMSD values (0.172 ± 0.024 nm for AChE–IPA), indicating minimal structural deviation after complex formation. In addition, increased RMSF values around loop regions adjacent to the binding pocket suggested enhanced local flexibility, particularly in IPA-bound systems, which may facilitate ligand adaptability. The average number of hydrogen bonds (2.11 per frame) further supported a stable and polar interaction environment. Compared to ABA, IPA formed more persistent polar and hydrophobic interactions, as reflected in both the higher hydrogen bond occupancy and increased local residue fluctuations. The enhanced local flexibility observed in RMSF profiles may be attributed to the conformational plasticity introduced by the isopentenyl chain. In the case of BChE, the absence of sustained hydrogen bonding combined with a significant reduction in RMSD and RMSF implies that IPA’s binding is predominantly governed by nonpolar interactions, likely involving π–stacking with aromatic residues such as Trp231 and Phe329. This supports the observed competitive inhibition pattern and the ligand’s ability to anchor within the catalytic gorge without disrupting overall protein compactness.

ABA is a sesquiterpenoid compound (C_15_H_20_O_4_) with a chiral center and structural moieties such as a free carboxyl group and conjugated double bonds that modulate its bioactivity. The presence of an α,β-unsaturated ketone system adjacent to the cyclohexene ring likely contributes to π–π stacking and hydrogen bond interactions with residues near the PAS of AChE. This is consistent with molecular docking results showing ABA’s preferential binding at peripheral rather than catalytic residues, supporting its experimentally observed non-competitive inhibition profile. MD simulations further corroborated this selective binding mode. ABA–AChE complexes exhibited lower RMSD values (0.162 ± 0.015 nm) than both IPA–AChE and apoenzyme controls, indicating tighter conformational restraint upon binding. RMSF values were also reduced (0.073 nm), particularly in loop regions near the PAS, suggesting diminished local flexibility consistent with a more rigid, localized interaction. The average hydrogen bond count remained low (0.84 per frame), and the absence of significant fluctuations in radius of gyration (2.295 ± 0.006 nm) reflected global structural stability without major conformational adaptation. These findings align with DFT-based electronic descriptors, which revealed a relatively narrow HOMO–LUMO gap and moderate electrophilicity, indicating chemical softness and the potential for selective interaction with polar microenvironments such as the PAS. Furthermore, ABA’s limited conformational adaptability and spatial restriction to the peripheral site may be explained by its relatively low dipole moment, in contrast to broader dual-binding ligands such as IPA. Collectively, the MD and electronic properties of ABA converge to support a model of PAS-restricted, allosteric inhibition of AChE.

The predicted pharmacokinetic profiles of IPA and ABA are consistent with their potential as CNS-active cholinesterase modulators. Both compounds exhibited excellent oral absorption and BBB permeability, with no predicted interaction with P-glycoprotein or hERG channels, suggesting adequate brain bioavailability and low cardiotoxic risk. These in silico findings provide preliminary ADMET evidence supporting the CNS-targeting potential of these compounds. IPA has received relatively little attention in the CNS pharmacology field, yet its molecular scaffold and predicted interactions suggest considerable potential as a cholinesterase modulator. Similarly, N6-isopentenyladenosine, a structural analog of IPA, has demonstrated neuroprotective and antioxidant effects in hippocampal neurons, and antiproliferative activity in glioma models [25,31], reinforcing the viability of this compound class for neurotherapeutic applications. In parallel, ABA has been shown to exert neuroprotective and anti-inflammatory effects in mammalian models, while exhibiting low cytotoxicity in neuronal cells at physiologically relevant concentrations [32,33,34,35].

Our computational results extend previous observations by providing a mechanistic basis for the potential of IPA and ABA as CNS-active cholinesterase modulators. Predicted properties such as oral bioavailability, BBB permeability, enzymatic selectivity, and binding stability support their candidacy for further pharmacological development. Based on these findings, subsequent studies should focus on evaluating their protective effects against neuronal injury, ability to modulate cholinergic signaling, and pharmacokinetic profiles including absorption, distribution, metabolism, and brain penetration in disease-relevant in vitro and in vivo models.

## 4. Materials and Methods

### 4.1. Reagents and Enzymes

IPA (≥90%), ABA (≥98.5%), SA (≥98.5%), JA (≥98.5%), IAA (98%), galantamine, AChE, BChE, 5,5′-dithio-bis-[2-nitrobenzoic acid] (DTNB), DMSO, HEPES, and acetylthiocholine iodide (ACTI) were purchased from Sigma-Aldrich (St. Louis, MO, USA). Trypsin, chymotrypsin, elastase, and their corresponding substrates were purchased from Sigma-Aldrich. All other chemicals and solvents used were of analytical grade.

### 4.2. Inhibitory Activity and Kinetic Analysis of Cholinesterases

Both AChE and BChE inhibitory activities were evaluated using the modified Ellman method under identical assay conditions, differing only in the enzyme (0.8 U/mL) and the corresponding substrate, acetylthiocholine iodide (ACTI) for AChE and butyrylthiocholine iodide (BTCI) for BChE [36]. First, AChE or BChE (0.8 U/mL) was added to a mixture containing 0.1 M sodium phosphate buffer (pH 8.0), DTNB (5 mM), and a test sample, followed by incubation at 37 °C for 15 min in 96-well microplates. Subsequently, the appropriate substrate, ACTI (500 µM) for AChE or BTCI (500 µM) for BChE, was added to initiate the reaction. Enzymatic hydrolysis was monitored at 405 nm using a Synergy H1 microplate reader (BioTek Instruments, Inc., Winooski, VT, USA), by detecting the formation of 5-thio-2-nitrobenzoic acid.

For kinetic analysis, varying concentrations of substrate were incubated with fixed concentrations of enzyme and multiple concentrations of the inhibitor. Dixon and Lineweaver–Burk plots were used to determine the mode of inhibition and the corresponding kinetic constants. Ki values were calculated from Dixon plots, whereas Km and Vmax were derived from Lineweaver–Burk plots using initial rate data. All calculations were performed using SigmaPlot™ v12.3 (Systat Software, Inc., San Jose, CA, USA).

### 4.3. Drug-Likeness and ADMET Analysis

Pharmacokinetic and safety profiles of isopentenyl adenine and abscisic acid were evaluated using ADMETlab 3.0 (https://admetlab3.scbdd.com/, accessed on 1 September 2025) [37]. SMILES notations were retrieved from PubChem (https://pubchem.ncbi.nlm.nih.gov/, accessed on 1 September 2025) [38]. Calculated descriptors included molecular weight, LogP, TPSA, number of rotatable bonds, hydrogen bond donors and acceptors, and Csp^3^ fraction [39].

The physicochemical profiles were visualized via heatmaps, providing a compact graphical representation in which normalized descriptor values are encoded as color intensities. Heatmaps were generated using Matplotlib v3.8.4 (Python Software Foundation, Beaverton, OR, USA) with normalized values to allow descriptor-level comparisons.

### 4.4. Density Functional Theory (DFT) Z Calculations

The electronic structures of IPA and ABA were investigated using DFT to evaluate their chemical reactivity [40]. Molecular structures were initially constructed and energy-minimized in Avogadro v1.2.0 (OpenChemistry, Pittsburgh, PA, USA) [41], followed by quantum chemical calculations using ORCA v6.0.1 (Max Planck Institute, Mülheim, Germany) [42]. Geometry optimizations were performed at the B3LYP/def2-SVP level, incorporating Grimme’s D3BJ dispersion correction and TightSCF convergence criteria. For improved precision in orbital energy determination, single-point energy calculations were carried out at the def2-TZVP level.

From the optimized wavefunctions, key quantum chemical descriptors were extracted, including HOMO and LUMO energies, energy gap (ΔE), dipole moment, zero-point energy, enthalpy, thermal energy, and Gibbs free energy. The HOMO–LUMO gap (ΔE = LUMO − HOMO) was used as an indicator of molecular stability and reactivity, while global reactivity descriptors such as chemical hardness (*η*), electronegativity (*χ*), and electrophilicity index (*ω*) were calculated using the following relationships:

$$\begin{array}{c} \eta =\frac{E_{LUMO}-E_{HOMO}}{2}\left(\mathrm{Chemical}\;\mathrm{hardness}\right)\\ \chi =-\frac{E_{LUMO}+E_{HOMO}}{2}\left(\mathrm{Electronegativity}\right)\\ \omega =\frac{\chi^{2}}{2\eta }\left(\mathrm{Electrophilicity}\;\mathrm{index}\right) \end{array}$$

### 4.5. Molecular Docking of Phytohormones with AChE and BChE

Molecular docking simulations were conducted to evaluate binding interactions of IPA and ABA with target enzymes. Three-dimensional structures of AChE (PDB ID: 4M0E) and BChE (PDB ID: 7AWG) were obtained from the RCSB Protein Data Bank (https://www.rcsb.org/, accessed on 18 September 2025) [43,44].

Protein preparation steps (ligand removal, hydrogen addition, Kollman charges) were performed in UCSF Chimera v1.18, and ligands were retrieved from PubChem in SDF format [45]. Conversion to PDB and structural minimization were carried out using Chimera, followed by PDBQT conversion via Open Babel v3.1.1 (Open Babel Project, Pittsburgh, PA, USA) [46]. Docking was executed with AutoDock Vina (v1.2.3) using the following grid settings:

- AChE: Grid center: x = −17.1, y = −42.41, z = 25.56; size: 17 × 17 × 17 Å
- BChE: Grid center: x = 137.09, y = 113.47, z = 41.85; size: 20 × 20 × 20 Å

Simulations were executed on macOS 15.6.1 (Apple M1, 8 GB RAM). Binding interactions (hydrogen bonds, π-stacking, and hydrophobic contacts) were analyzed using PyMOL v3.0 (Schrödinger, LLC, New York, NY, USA) and BIOVIA Discovery Studio Visualizer v2021 (BIOVIA, San Diego, CA, USA).

### 4.6. Molecular Dynamics

To evaluate the conformational stability of ligand-protein complexes, MD simulations were carried out using GROMACS v2023.3 (University of Groningen, Groningen, Netherlands) [47]. The CHARMM27 force field was applied for protein parameters, and ligand topologies were generated via SwissParam (https://old.swissparam.ch/, accessed on 14 October 2025) [48].

Each complex was solvated in a triclinic TIP3P water box and neutralized with 0.1 M Na^+^/Cl^−^. Energy minimization was performed using steepest descent (50,000 steps), followed by equilibration:

- NVT ensemble: 100 ps at 300 K (Berendsen thermostat)
- NPT ensemble: 100 ps at 1 bar (Parrinello–Rahman barostat)

Production simulations of 100 ns were conducted under constant pressure and temperature conditions.

To assess the dynamic behavior of the ligand–enzyme complexes, post-simulation analyses focused on several structural descriptors. RMSD was used to monitor overall stability, while RMSF provided insights into local flexibility at the residue level. Rg values reflected the compactness of the complex throughout the trajectory, and hydrogen bond counts helped track the persistence of intermolecular interactions. All analyses were visualized using XMGrace v5.1.25 (Grace Development Team, Portland, OR, USA), and temporal trends in binding stability were evaluated over the 100 ns simulation period [49].

### 4.7. MM/PBSA Analysis

Binding free energies (ΔG_bind_) were estimated using the MM/PBSA approach to assess the strength of protein–ligand interactions. Analyses were performed on trajectories obtained from molecular dynamics simulations using the gmx_MMPBSA package, compatible with GROMACS 2023.3 [50], together with the corresponding topology and parameter files. The binding free energy was determined according to the equation:

ΔG_bind_ = ΔG_complex_ − (ΔG_protein_ + ΔG_ligand_)

where ΔGc_omplex_, ΔG_protein_, and ΔG_ligand_ denote the free energies of the complex, receptor, and ligand, respectively. The total binding energy comprised contributions from vacuum interaction energy, polar solvation energy, and nonpolar solvation energy. Subsequent post-processing and energy decomposition were carried out using gmx_MMPBSA_ana, enabling thermodynamic interpretation of ligand binding behavior observed in docking and molecular dynamics simulations.

## 5. Conclusions

The present study identified IPA and ABA, two phytohormones abundant in marine organisms, as previously unrecognized cholinesterase modulators with potential CNS pharmacological relevance. Through a combined experimental and computational approach, we demonstrated that IPA exhibits dual-site interaction with AChE and competitive inhibition of BChE, while ABA acts as a non-competitive, PAS-restricted AChE inhibitor. These inhibitory profiles were supported by quantum chemical descriptors and molecular dynamics simulations, revealing distinct binding behaviors driven by molecular polarity, structural features, and interaction stability. Notably, the purine-based scaffold of the IPA showed strong complementarity to the catalytic gorge of BChE and sustained interactions across the active site of AChE, suggesting multi-site compatibility. Conversely, the spatially restricted binding of ABA within the PAS, consistent with its electronic softness and low dipole moment, suggests a mechanism of allosteric AChE inhibition. Predicted pharmacokinetic properties further supported adequate BBB permeability, oral bioavailability, and low toxicity risks for both compounds. Collectively, these findings highlight the potential of IPA and ABA as lead candidates for cholinesterase-targeted intervention in neurodegenerative disorders. Future in vitro and in vivo studies will be necessary to validate their neuroprotective efficacy and clarify their disease-modifying capacity.

## Figures and Tables

**Table 1:** Physicochemical descriptors of phytohormones and galantamine.

Descriptor	Desired Value	Compounds					
		IPA	ABA	SA	JA	IAA	Galantamine ^1^
MW (g/mol) ^2^	<500	203.25	264.32	138.12	210.27	175.18	287.37
LogP ^3^	<5	1.73	2.25	2.26	3.04	1.02	1.53
TPSA(Å^2^) ^4^	≤140	66.49	74.60	57.53	83.58	63.32	41.93
H-bond donor	≤5	2	2	1	2	2	1
H-bond acceptor	≤10	4	3	3	4	3	4
Drug-likeness	0: excellent	0	0	0	0	0	0
Rotatable bonds	-	3	3	1	4	3	4
Rings	-	2	1	1	1	2	3
Heavy atoms	-	15	19	11	15	13	20
Fraction Csp3 ^5^	-	0.3	0.47	0.08	0.65	0.24	0.55

^1^ Galantamine was used as a positive control; ^2^ Molecular weight; ^3^ Lipophilicity; ^4^ Topological polar surface area; ^5^ Fraction of sp^3^ carbons.

**Table 2:** Cholinesterase inhibitory activity of selected active compounds.

Compounds	AChE			BChE		
	IC_50_ (µM) ^1^	Ki Value ^2^	Inhibition Type	IC_50_ (µM) ^1^	Ki Value ^2^	Inhibition Type
IPA	40.52 ± 0.71	46.8	Non-competitive	25.81 ± 0.05	13.0	Competitive
ABA	76.29 ± 2.94	76.3	Non-competitive	>100	-	-
Galantamine ^2^	1.72 ± 0.13	-	Competitive	12.21 ± 0.55	-	Competitive

^1^ Values are expressed as mean ± standard deviation (SD) based on at least three independent experiments. ^2^ “–” indicates data not determined or not applicable.

**Table 3:** Inhibitory activity against serine proteases for assessing target specificity.

Compounds	Conc. (µM)	Trypsin (%)	Chymotrypsin (%)	Elastase (%)
IPA	50	3.80 ± 1.77	4.72 ± 0.79	4.74 ± 2.80
	100	4.73 ± 1.47	6.67 ± 2.36	5.56 ± 1.76
ABA	50	5.93 ± 1.27	12.26 ± 2.94	5.28 ± 1.38
	100	4.63 ± 2.54	17.74 ± 0.53	5.46 ± 0.95

**Table 4:** Molecular docking results of isopentenyl adenine and abscisic acid with AChE and BChE.

Enzymes	Resolution	Ligands	Free Energy (kcal/mol)	No. of H-Bond	Residues	Bond Distance (Å)	Other Interactions
AChE (PDB: 4M0E)	2.00 Å	IPA	−7.83	4	Tyr124 Ser203 Gly121 Gly122	2.41 2.90 2.92 2.92	Trp86, His447 (π-π T-shaped) Tyr341, Trp286 (π-alkyl)
		ABA	−7.04	2	Tyr124 Tyr341	2.30 2.22	-
		Galantamine	−7.39	1	Phe295	2.47	Trp286, Phe297 (π-alkyl)
BChE (PDB: 7AWG)	2.00 Å	IPA	−7.26	1	Leu286	2.36	Trp231 (π-π T-shaped) Phe329 (π-sigma, π-π T-shaped, π-alkyl) Leu286, Phe357 (π-alkyl) Val393 (alkyl)
		Galantamine	−9.01	4	Asn83 Gly116 Trp82 Glu197	3.72 3.60 3.60 3.45	Trp82 (π-donor hydrogen bond, π-π stacked) His438 (π-alkyl)

**Table 5:** Prediction of pharmacokinetic and safety profiles of isopentenyl adenine and abscisic acid by ADMETlab.

Property	Desired Value	IPA	ABA
Human intestinal absorption (%)	Excellent	Excellent	Excellent
Substrate of P-glycoprotein (P-gp)	No	No	No
Inhibitor of P-gp	No	No	No
Permeability of BBB	Yes	Yes	Yes
hERG inhibition (cardiotoxicity)	No	No	No

**Table 6:** Binding free energy components of IPA, ABA, and GAL complexes with AChE and BChE.

Complex	VDWAALS	EEL	EGB	ESURF	GGAS	GSOLV	TOTAL
IPA–AChE	−17.91	−22.2	26.21	−2.80	−40.11	23.40	−16.71
ABA–AChE	−16.48	−14.31	20.24	−2.51	−30.79	17.73	−13.06
GAL–AChE	−29.57	−238.64	247.90	−3.82	−268.21	244.08	−24.13
IPA–BChE	−30.16	−8.28	21.32	−3.54	−38.44	17.78	−20.66
GAL–BChE	−33.71	−130.52	143.37	−4.13	−164.23	139.24	−25.00

ΔVDWAALS: van der Waals energy; ΔEEL: electrostatic energy; ΔEGB: polar solvation energy calculated using the Generalized Born model; ΔESURF: nonpolar solvation energy; ΔGGAS: sum of van der Waals and electrostatic energies; ΔGSOLV: sum of polar and nonpolar solvation energies; ΔTOTAL: total binding free energy (ΔGGAS + ΔGSOLV).

## Data Availability

The original contributions presented in this study are included in the article/Supplementary Material. Further inquiries can be directed to the corresponding author

## Supplementary Materials

The following supporting information can be downloaded at: https://www.mdpi.com/article/10.3390/md24010035/s1, Figure S1: DFT-calculated HOMO–LUMO energy levels and orbital distributions of IPA, ABA, and galantamine; Table S1: Electronic reactivity descriptors of isopentenyl adenine and abscisic acid.

## Abbreviations

The following abbreviations are used in this manuscript:

AD	Alzheimer’s disease
Aβ	amyloid-beta
AChE	acetylcholinesterase
BChE	butyrylcholinesterase
PAS	peripheral anionic site
PS-1	presenilin-1
BBB	blood–brain barrier
ChEIs	cholinesterase inhibitors
CNS	central nervous system
CAS	catalytic active site
QSAR	quantitative Structure–Activity Relationship
IPA	isopentenyl adenine
ABA	abscisic acid
SA	salicylic acid
JA	jasmonic acid
IAA	indole-3-acetic acid
MW	molecular weight
LogP	lipophilicity
TPSA	topological polar surface area
Csp^3^	fraction of sp^3^-hybridized carbons
ADMET	absorption, distribution, metabolism, excretion, and toxicity
DFT	density functional theory
HOMO	highest occupied molecular orbital
LUMO	lowest unoccupied molecular orbital
Ki	inhibition constants
P-gp	P-glycoprotein
MD	molecular dynamics
RMSD	root mean square deviation
RMSF	root mean square fluctuation
Rg	radius of gyration
DTNB	5,5′-dithio-bis-[2-nitrobenzoic acid]
ACTI	acetylthiocholine iodide
BTCI	butyrylthiocholine iodide
