# Supplementary material for: Structural and Mechanistic Insights into Dual Cholinesterase Inhibition by Marine Phytohormones
Source: Mar Drugs. 2026 Jan 9;24(1):35. doi: 10.3390/md24010035 (PMC12842749; doi:10.3390/md24010035)

Figure S1. DFT-calculated HOMO–LUMO energy levels and orbital distributions of IPA, ABA, and galantamine

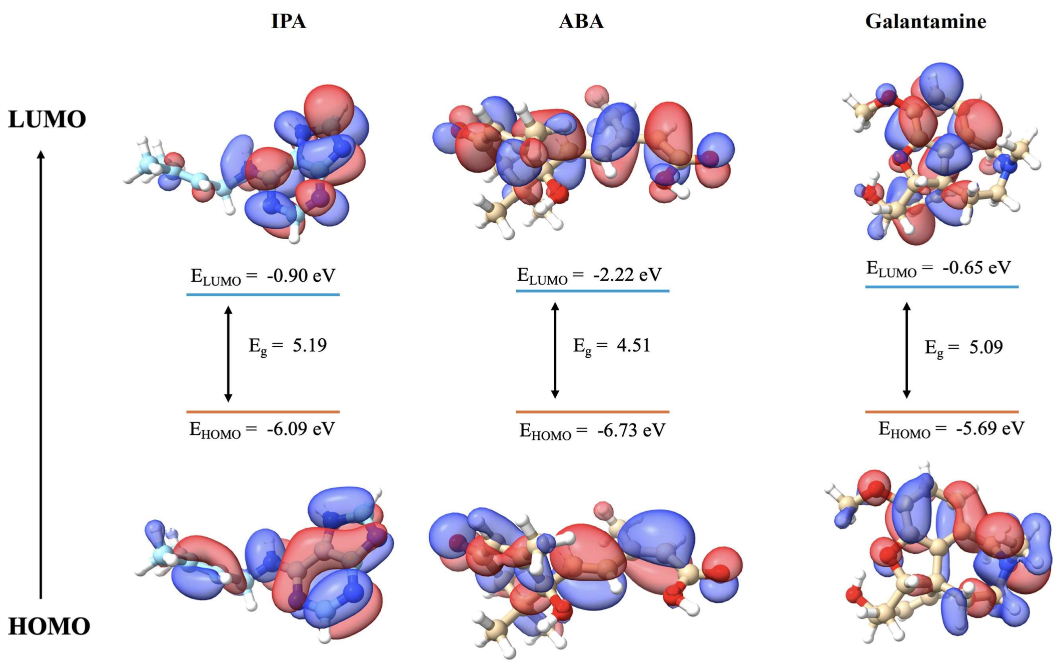

Supplement: Supplementary file 1 [file marinedrugs-24-00035-s001.zip › marinedrugs-4014052-Figure S1.pdf]
