# Supplementary material for: Structural and Mechanistic Insights into Dual Cholinesterase Inhibition by Marine Phytohormones
Source: Mar Drugs. 2026 Jan 9;24(1):35. doi: 10.3390/md24010035 (PMC12842749; doi:10.3390/md24010035)
Supplement: Supplementary file 1 [file marinedrugs-24-00035-s001.zip › marinedrugs-4014052-Table S1.pdf]

Table S1. Electronic reactivity descriptors of isopentenyl adenine and abscisic acid.

Compounds	Electronic descriptors							Thermodynamic descriptors		
	Energy gap (eV[1])	Dipole moment (Debye)	Electrophilicity index (ω, eV)	Hardness (η, eV)	Softness (S, eV⁻¹)	Electronegativity (χ, eV)	Mean energy (μ, eV)	Total energy (Eh[3])	Gibbs Free energy (Eh)	Optimization energy (Eh)
IPA	5.19	7.53	2.35	2.6	0.39	-3.5	3.5	-661.52	-661.58	-661.77
ABA	4.51	2.25	4.45	2.26	0.44	-4.48	4.48	-883.02	-883.07	-883.34
Galantamine	5.09	2.04	2	2.52	0.4	-3.17	3.17	-939.2	-939.27	-939.58

[1] Energy gap calculated as lowest unoccupied molecular orbital (LUMO) - highest occupied molecular orbital (HOMO); [2] Electronvolts(eV); [3] Energies in Hartree (Eh) obtained from DFT calculations using B3LYP/def2-SVP for geometry optimization and def2-TZVP for single-point energy.
